# Growth and Physiological Performance of a Coastal Species *Trifolium fragiferum* as Affected by a Coexistence with *Trifolium repens*, NaCl Treatment and Inoculation with Rhizobia

**DOI:** 10.3390/plants10102196

**Published:** 2021-10-16

**Authors:** Kārlis Dūmiņš, Una Andersone-Ozola, Ineta Samsone, Didzis Elferts, Gederts Ievinsh

**Affiliations:** 1 Department of Plant Physiology, Faculty of Biology, University of Latvia, 1 Jelgavas Str., LV-1004 Riga, Latvia; karlis.dumins@gmail.com (K.D.); una.andersone-ozola@lu.lv (U.A.-O.); ineta.samsone@inbox.lv (I.S.); 2 Department of Botany and Ecology, Faculty of Biology, University of Latvia, 1 Jelgavas Str., LV-1004 Riga, Latvia; didzis.elferts@lu.lv

**Keywords:** NaCl, physiological performance, rhizobia, species coexistence, *Trifolium fragiferum*, *Trifolium repens*

## Abstract

The aim of the present study was to analyze the growth and physiological performance of two coexisting species, *Trifolium fragiferum*, and *Trifolium repens*, under the effect of NaCl and rhizobial symbiosis. Seeds of *T. fragiferum* and *T. repens* were collected from populations in the wild, and plants were cultivated in an automated greenhouse, two plants per container. Three basic types of planting were performed: (1) both plants were *T. fragiferum* (single species), (2) one *T. fragiferum* and one *T. repens* (species coexistence), (3) both plants were *T. repens* (single species). For every basic type, three subtypes were made: (1) non-inoculated, (2) inoculated with rhizobia taken from *T. fargiferum*, (3) inoculated with rhizobia taken from *T. repens*. For every subtype, half of the containers were used as control, and half were treated with NaCl. Shoot fresh mass of plants was significantly (*p* < 0.001) affected by species coexistence, inoculant, and NaCl. Three significant two-way interactions on plant shoot growth were found: between species coexistence and NaCl (*p* < 0.001), inoculant and species (*p* < 0.05), and NaCl and species (*p* < 0.001). A significant three-way interaction between inoculant, NaCl, and species (*p* < 0.001) indicated different responses of shoot growth of the two species to inoculant type and NaCl. NaCl treatment was an important factor for *T. fragiferum*, resulting in better growth in conditions of species coexistence, but the positive effect of bacterial inoculant was significantly more pronounced. A decrease in peroxidase activity in leaves was a good indicator of relative NaCl tolerance, while the absence/presence of rhizobial inoculation was reflected by changes in leaf chlorophyll concentration and photochemical activity of photosystem II. It can be concluded that interaction between biotic and abiotic factors affected the outcome of the coexistence of the two *Trifolium* species. Distribution of *T. fragiferum* in sea-affected habitats seems to be related to a higher competitive ability with allied species at increased substrate salinity, based on better physiological salinity tolerance.

## 1. Introduction

Species rarity is a complex ecological problem [1]. Considering species habitat specificity and geographical range of distribution, functional aspects of plant species’ rarity are extremely important. One promising approach can be the comparison of closely related species growing in the same habitat [2]. However, competition between closely related plant species often is a neglected aspect in ecophysiological studies. When differences in rarity between the coexisting species are evident, more rare species most likely represent a reflection of less competitive and/or adaptive ability in comparison to the more common ones. In particular, it is argued that species on the border of distribution have suppressed adaptive potential resulting in decreased fitness [3]. From a physiological point of view, competitive ability and adaptive potential can be assessed not only through measuring differences in persistence and growth, but also by means of determining variation in physiological and biochemical parameters of adaptive nature.

*Trifolium fragiferum* L. (strawberry clover) is a perennial stoloniferous clonal legume (Fabaceae) species native to Europe, Asia, and Africa. The species is relatively tolerant to flooding and moderate soil salinity [4], it is sometimes classified as mesohydrohalophilic euhalophyte [5]. In Northern Europe, *T. fragiferum* is relatively rare and exclusively associated with an endangered habitat Baltic coastal meadow [6]. As an endangered species, *T. fragiferum* is included in the Regulations of the Cabinet of Ministers of Latvia No. 396, 2000 [7]. Another perennial clover species, *Trifolium repens* L. (white clover), is more widely distributed within the moist temperate, Mediterranean, and cool subtropical zones. The species has been characterized as possessing relatively low tolerance to salinity [8] together with a good frost tolerance allowing for distribution in cold climates [9]. Both species have commercial importance as forage legumes.

In Latvia, both *T. fragiferum* and *T. repens* are present in a salt-affected wet meadow near Lake Liepajas, which is Natura 2000 birds and habitats directives site (“Liepajas ezers” LV0507500) pointing to the interaction between the two species in natural conditions. A relationship between *T. repens* and *T. fragiferum* was assessed a relatively long time ago in natural field conditions [10]. It was found that *T. repens* developed a larger leaf area than *T. fragiferum* due to the higher number of leaves produced. More recently, clonal growth performance as an evolutionary priority over flowering was studied in non-competitive experiments with *T. repens* and *T. fragiferum* [11].

Environmental factors can affect the physiological performance of plants directly or through the interactive effects on plant competition. Increased soil salinity due to inundation with seawater is a significant environmental factor in coastal meadows and coastal marshes [12]. Therefore, it is logical to assume that substrate salinity can be viewed as a main putative abiotic factor modifying the interaction between individuals of *T. fragiferum* and *T. repens*. In addition, symbiosis with nitrogen-fixing rhizobia has been described as an important constituent in the mineral nutrition of *Trifolium* species [13]. The outcome of the interaction between closely related species in conditions of high substrate salinity could be affected by the efficiency of symbiotic nitrogen fixation [14]. Resistance of rhizobial bacteria to soil salinity may differ from that of their host, moreover, the response of symbiotic nodule to salinity can be different from both individual responses [15,16].

Plant physiological performance as a putative indication to adaptations to environmental constraints or competitive ability can be characterized by analysis of a wide range of plant responses, including growth, photosynthesis-related parameters, enzymatic oxidative, and antioxidative processes, as well as biochemical indicators of cellular damage. Non-destructive characterization of the physiological performance of wild plants has been a challenge within the last decades. Thus, fast continuous chlorophyll *a* fluorescence analysis has been successfully used as a reliable and sensitive method in salinity response studies of coastal marsh species [17] or drought stress tolerance assessment [18]. Other photosynthesis-related traits, including leaf chlorophyll concentration, have become useful characteristics for overall plant responses to various environmental factors [19,20]. Non-respirative enzymatic oxidative activity in plants is often characterized by means of peroxidase and/or polyphenol oxidase activity. It can be related to plant defense responses both through the synthesis of antioxidants and protective substances as well as contribution to enzymatic antioxidative as well as prooxidative systems [21,22,23]. Measurement of electrolyte leakage from plant tissues has been frequently used to assess the degree of damage to cell membranes [24,25]. Another characteristic of oxidative stress-related peroxidative membrane damage is tissue malondialdehyde (MDA) concentration, usually measured by means of analyzing the concentration of thiobarbituric acid-reactive substances (TBARS) [26].

Ion relationships are especially important for salinity-affected plants, as differences in ion compartmentation can result in significant differences in salinity tolerance [27]. For example, K, the only non-structural essential mineral nutrient in plants, is involved in the activation and stabilization of enzymes, membrane functioning, osmotic maintenance [28]. Most importantly, K has been shown to be involved in the mediation of plant adaptive responses to a range of environmental factors, including salinity, through the maintenance of cellular K homeostasis [29,30]. Chemical similarity between Na and K allows predicting the utilization of Na instead of K for several important metabolic functions [31]. For example, it was shown that Na can near completely replace the osmotic function of K in the vacuole in beet plants [31] and several halophyte species [32]. 

Species interactions have been assessed in field conditions through some experimental manipulation leading to changes in resource availability, and usually comparing responses in respect to the natural gradient of certain environmental factors [33,34] as well as through studies in controlled conditions [35,36]. Recently, more information has become available on the role of soil microorganisms in predicting the outcome of plant species coexistence through multifaceted interactions [37,38] and this also gained a broader theoretical perspective [39]. As field experiments are not suitable for rare plant species in specially protected sites, the aim of the present study was to assess the growth and physiological performance of two coexisting species, *T. fragiferum* and *T. repens*, in controlled conditions, as affected by NaCl and rhizobial symbiosis. The hypothesis tested was that *T. fragiferum* could outcompete *T. repens* only in conditions of increased substrate salinity. In addition, it was asked: what is the role of nitrogen-fixing rhizobial symbiosis in the outcome of the coexistence between the species, performing artificial inoculation of plants grown in sterile soil with rhizobia?

## 2. Materials and Methods

### 2.1. Field Measurements

Field measurements and sampling were performed in a salt-affected wet meadow near Lake Liepajas (Natura 2000 birds and habitats directives site “Liepajas ezers” LV0507500). Three plots with coexisting genets of *T. fragiferum* and *T. repens* were selected approximately 200 m from a coastline within a diameter of 50 m (56°29′37″ N, 21°01′40″ E). Soil salinity was measured as electrical conductivity (EC) at each plot in triplicate using an HH2 moisture meter equipped with a WET-2 sensor (Delta-T Devices, Burwell, UK). Lake water EC, Na^+^, and K^+^ concentrations were measured in triplicate using LAQUAtwin (Horiba, Kyoto, Japan) compact meters B-771, B-722, and B-731, respectively. Small tissue samples consisting of randomly selected plant parts (stolons, leaf petioles, leaves, flower stalks, flowers) of both species were collected at each plot. The samples were taken to the laboratory, dried in an oven at 60 °C for 72 h, and used for the analysis of Na^+^ and K^+^ concentrations as described further (2.5).

### 2.2. Plant Material, Growth Conditions and Treatments

Seeds of *T. fragiferum and T. repens* were collected at the end of August 2016 from plants growing in a salt-affected wet meadow near Lake Liepajas, Latvia. The study was performed during the winter season in partially controlled conditions. After drying at room temperature for two weeks, the seeds were stored at 4 °C. Seeds were scarified by hand using a scalpel and imbibed in sterile deionized water for 4 h, then they were sown in sterile plastic tissue culture containers filled with 1 cm autoclaved commercial garden soil (Biolan, Eura, Finland) mixed with sterile deionized water. Containers were placed in a growth cabinet with a 16 h photoperiod (100 µmol m^−2^ s^−1^) at 23 °C. After the appearance of the first two true leaves, seedlings were transplanted to sterilized 200 mL plastic containers filled with autoclaved commercial garden soil (Biolan, Eura, Finland) mixed with sterile deionized water (200 mL water per 1 L soil). Containers were kept in sterilized 48 L plastic boxes closed with lids in an experimental automated greenhouse (HortiMax, Maasdijk, Netherlands) with supplemented light from Master SON-TPIA Green Power CG T 400 W (Philips, Amsterdam, Netherlands) and Powerstar HQI-BT 400 W/D PRO (Osram, Munich, Germany) lamps (380 µmol m^−2^ s^−1^ at the plant level), 16 h photoperiod, day/night temperature 23/15 °C, relative air humidity 60 to 70% to ensure optimum growth conditions of plants. 

After three weeks, plants were transplanted in 1.2 L plastic containers filled with autoclaved commercial garden soil (Biolan, Eura, Finland), two plants per container. Three basic types of planting were performed: (1) both plants were *T. fragiferum* (single species, TFss), (2) one *T. fragiferum* and one *T. repens* (species coexistence, TFsc and TRsc), (3) both plants were *T. repens* (single species, TRss). For every basic type, three subtypes were made: (1) non-inoculated (−i), (2) inoculated with rhizobia taken from *T. fargiferum* (+iTF), (3) inoculated with rhizobia taken from *T. repens* (+iTR). For every subtype, half of the containers were used as control, and half were treated with NaCl as described further. As a result, 18 treatments were established (Table 1) with five containers per treatment.

Bacterial inoculant was prepared from single individual plants of the respective species found at the same site where the seeds were collected. Plant roots were surface sterilized in 50% commercial bleach (ACE, Procter & Gamble, Warszawa, Poland) for 15 min and rinsed thoroughly with sterile deionized water four times. Nodules were detached and bacterial cells were isolated from the nodules according to the standard procedure on yeast-mannitol agar [40]. For inoculation, 6 mL per container of the respective bacterial suspension (about 10^9^ bacterial cells per mL) was used, supplying 1 mL of bacterial suspension in six points evenly over the surface of the substrate. All procedures were performed in a laminar box using sterilized instruments and material. 

After transplanting and bacterial inoculation, plants were cultivated for two weeks in the greenhouse in conditions as described above. Individual containers were randomly placed in a greenhouse and were repositioned twice a week. Plants were irrigated with deionized water twice a week not allowing a decrease in substrate moisture lower than 60% water holding capacity. An individual watering system of containers was used to decrease possible contamination with rhizobial bacteria in the early stages of the experiment, where each container had a plastic plate under it for accumulation of excessive water. Substrate water content was monitored with an HH2 moisture meter equipped with a WET-2 sensor (Delta-T Devices, Burwell, UK). Starting from the third week after transplanting, respective containers (treatment +NaCl) were irrigated with an increasing concentration of NaCl. Once a week, +NaCl containers were treated with 50 mL NaCl solution (starting with 25 mM, followed by 50, 100, 200, 300, and 400 mM), but –NaCl plants received 50 mL deionized water. 

Plants were irrigated with deionized water when necessary and were fertilized biweekly with a Kristalon Green Label fertilizer (NPK 18-18-18 with micronutrients; Yara International, Oslo, Norway) solubilized in deionized water (150 g L^−1^), with 5 mL of stock solution per L, 120 mL of the final fertilizer per container. Nitrogen was present both in the form of nitrate-N (9.8%) and ammonia-N (8.2%).

### 2.3. Assessment of Substrate Electrical Conductivity and Physiological Measurements

Substrate electrical conductivity was measured three times during the plant cultivation (on week 2, 4, and 6) with an HH2 moisture meter equipped with a WET-2 sensor (Delta-T Devices, Burwell, UK). For every container, four separate measurements were performed.

Measurements of leaf chlorophyll concentration and chlorophyll *a* fluorescence in leaves were started one week after the start of the NaCl treatment (week 1) and were performed weekly for the next five weeks (week 6). Three containers per treatment were randomly selected for analysis each week. For every individual, three fully grown leaves were selected for chlorophyll concentration analysis and another three for analysis of chlorophyll *a* fluorescence. Chlorophyll concentration was measured by a chlorophyll meter CCM-300 (Opti-Sciences, Hudson, NH, USA). Chlorophyll *a* fluorescence was measured in leaves dark-adapted for at least 20 min by Handy PEA fluorometer (Hansatech Instruments, King’s Lynn, UK). For characterization of photochemical activity, chlorophyll *a* fluorescence parameter Performance Index (total) was used. Performance Index is a complex indicator of photochemical efficiency combining three function-related (trapping of absorbed exciton, electron transport between the photosystems, reduction of end-electron acceptors) and structure-related (antenna chlorophyll per reaction center chlorophyll) parameters [41].

### 2.4. Plant Harvest

The effect of, as described below. The experiment was terminated on week 7. Plant shoots were cut and *T. fragiferum* and *T. repens* individuals from treatment combinations with species coexistence were separated according to leaf morphological features. Roots were not used and root parameters were not measured as it would be technically impossible to separate roots of both individuals from different species in species coexistence conditions. Total shoot biomass was measured and samples were collected for biochemical analyses (except for ion measurement). Each sample consisted of several leaf blades or petioles per species per treatment. Plant material from different containers were pooled to obtain the necessary amount (about 1 g fresh mass) per treatment per replication. Except for material for electrolyte leakage measurement, plant tissues were frozen in liquid N_2_ and stored at –20 °C until analysis. 

Plant shoots were dried in an oven at 60 °C for 72 h and then dry mass was measured. The dried material was used for the analysis of ion concentration in different plant parts.

### 2.5. Measurement of Electrolyte Leakage and Biochemical Parameters

Three replicates per treatment per species were used for the analysis of all biochemical parameters and electrolyte leakage. Plant material from every two containers were pooled, and material from the last fifth container was used as a third replicate.

Dried tissue samples (0.2 g) were used for estimation of Na^+^ and K^+^ concentration [42]. Tissues were ground with mortar and pestle to a fine powder and 10 mL of deionized water was added. After filtration through nylon mesh cloth (No. 80) homogenate was used for measurement of ion concentration by LAQUAtwin (Horiba, Kyoto, Japan) compact meters B-722 (Na^+^) and B-731 (K^+^). 

Malondialdehyde (MDA) content was estimated by measurement of the concentration of thiobarbituric acid-reactive substances (TBARS) according to Aref et al. (2016) [43]. Briefly, frozen plant material was ground with mortar and pestle to a fine powder and extracted with 0.1% trichloroacetic acid (10 mL g^−1^ FM), centrifuged at 12,000× *g* at 4 °C. To 1 mL of supernatant 4 mL of 0.5% thiobarbituric acid in 20% trichloroacetic acid was added and heated for 30 min at 90 °C. After rapid cooling in an ice bath, the mixture was centrifuged at 10,000× *g* for 5 min at room temperature and absorbance was measured at 532 and 600 nm. Results were expressed on a dry mass basis. 

Relative electrolyte leakage was measured as described previously [44]. Leaf discs (15 per analysis, 0.5 cm^2^ each) were prepared from fresh leaves, rinsed with deionized water three times, and immersed in tubes with 10 mL deionized water for 22 h at room temperature. Then, the electrical conductivity of the solution was measured using LAQUAtwin compact conductivity meter B-771 (Horiba Scientific, Kyoto, Japan). Tubes were incubated in a water bath at 80 °C for 2 h, cooled to room temperature, and the final conductivity was measured. 

Enzyme activities were measured using a frozen plant material [45]. Briefly, for preparation of extracts, plant tissue was frozen in liquid nitrogen, ground with mortar and pestle to a fine powder, and extracted with 25 mM HEPES/KOH buffer (pH 7.2) containing 1 mM EDTA, 3% polyvinylpolypirrolidone, 0.8% Triton X-100 (5 mL of buffer per g FM) for 15 min. After centrifugation at 15,000× *g* at 4 °C for 20 min, the supernatant was used for measurement of enzyme activity. Peroxidase activity was measured using guaiacol and H_2_O_2_ as substrates, and polyphenol oxidase activity using catechol. 

### 2.6. Data Analysis

For all dependent variables, homogeneity of variance was tested using the Levene test. If a violation was observed then variables were log-transformed. Analysis of variance (ANOVA) was used to test the main effects as well as all two-, three-, and four-way interactions of factors: species, NaCl, inoculant, and species coexistence, if there were enough observations in each combination. A separate ANOVA model was developed for each dependent variable. To estimate the effect size on shoot biomass, bias-corrected estimator ω^2^ was used. For significant variables, Tukey HSD tests were used as a post hoc test to determine significant differences between factor levels. All analyses were performed using software R 3.4.1 [46].

## 3. Results

### 3.1. Field Measurements

Average soil EC in plots with coexisting *T. fragiferum* un *T. repens* plants was 238.7 ± 8.8 mS m^−1^. The EC of the lake water near the sampling site was 4.3 ± 0.6 mS cm^−1^, and it contained 2.13 ± 0.03 g L^−1^ Na^+^ and 0.095 ± 0.003 g L^−1^ K^+^. 

**Figure 1:**
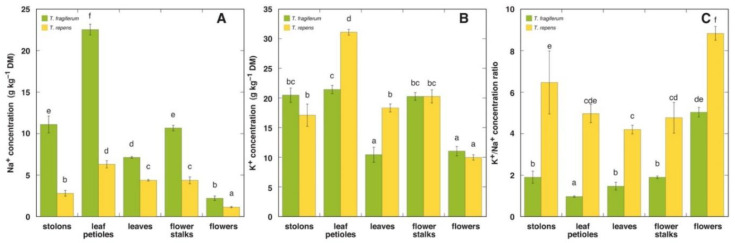
Na^+^ (**A**) and K^+^ (**B**) concentration, and K^+^/Na^+^ concentration ratio (**C**) in different parts of coexisting *T. fragiferum* and *T. repens* plants in field conditions of a salt-affected wetland. Different letters indicate significant differences between the treatments (*p* < 0.05).

Coexisting plants of the two allied *Trifolium* species showed significant differences in Na^+^ accumulation when growing in a natural saline-affected wetland habitat (Figure 1A). All parts of *T. fragiferum* plants had significantly more Na^+^ than these of *T. repens*. For both species, the highest Na^+^ concentration was in leaf petioles, significantly decreasing in stolons, flower stalks, and leaves; it was lowest in flowers. The K^+^ concentration in different parts of the two species was relatively similar (Figure 1B). Leaf petioles and leaves of *T. repens* had significantly higher K^+^ concentrations than the respective parts of *T. fragiferum*. The K^+^ concentration was significantly lower in flowers of both species in comparison to other parts. In general, the K^+^:Na^+^ concentration ratio was significantly higher in respective parts of *T. repens* in comparison to *T. fragiferum* (Figure 1C). However, the concentration ratio in flowers of *T. fragiferum* was significantly higher than in other parts.

### 3.2. Substrate Salinity

In controlled conditions, substrate electrical conductivity was monitored to document changes in soluble salt concentration due to mineral uptake and fertilization during growth as well as the rise in substrate NaCl concentration due to the gradual increase in salt concentration in a watering solution (Table 2). As expected, the gradual decrease in substrate EC in treatments without NaCl reflected the general depletion of mineral elements in spite of regular fertilization indicating that plants indeed were in nutrient-limiting conditions. According to the results of ANOVA, substrate EC was significantly affected by all single experimental factors (species coexistence, bacterial inoculant, NaCl, and time), as well as by interaction between species coexistence and inoculant, species coexistence, and NaCl, inoculant and NaCl, inoculant and time, as well as NaCl and time (Table 3).

### 3.3. Morphological Parameters

**Figure 2:**
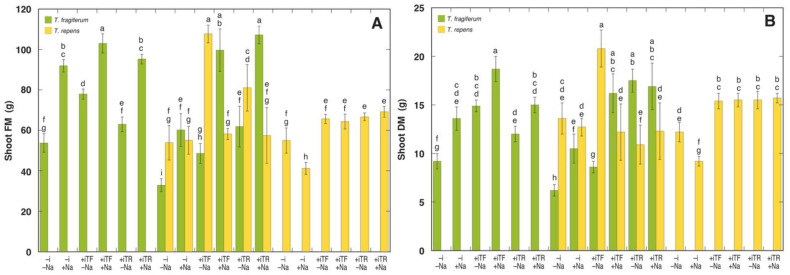
Shoot fresh mass (**A**) and dry mass (**B**) of *T. fragiferum* and *T. repens* plants grown in initially sterile soil. Data are the means from five replicates ± SE, the results are expressed on a single plant basis. –i, without inoculant; +iTF, with inoculant isolated from *T. fragiferum*; +iTR, with inoculant isolated from *T. repens*; –Na, no NaCl added; +Na, NaCl up to 400 mM. Different letters indicate significant differences between the treatments (*p* < 0.05).

The main morphological characteristics of plants of the two *Trifolium* species as affected by the experimental variables are shown in Figure 2 and Table 4. A fresh shoot mass of individual plants of *T. fragiferum* ranged from 32.9 (species coexistence without inoculant without NaCl) to 107.2 g (species coexistence with inoculant from *T. repens* and NaCl treatment), and for *T. repens* it ranged from 41.2 (single species without inoculant with NaCl treatment) to 107.7 g (species coexistence with inoculant from *T. fragiferum* without NaCl treatment) (Figure 2A). The lowest shoot dry mass for *T. fragiferum* was also for the treatment with species coexistence without inoculant without NaCl (6.2 g) but the highest was in the case of single species with inoculant from *T. fragiferum* with NaCl treatment (18.7 g) (Figure 2B). For *T. repens*, the lowest and highest shoot dry mass values were for the same treatments as in the case of shoot fresh mass (9.2 and 20.8 g, respectively). The lowest and highest number of leaves were in treatments with respectively lower and higher shoot fresh mass for both species (Table 4). The same relationship was evident for total stolon length in *T. fragiferum*, while in *T. repens* the highest stolon length corresponded to the highest shoot mass and number of leaves, but the lowest length was in the treatment with species coexistence with inoculant from *T. repens* and with NaCl treatment. The number of stolons varied from 32.5 (species coexistence with inoculant from *T. repens* without NaCl) to 77.0 (species coexistence with inoculant from *T. repens* with NaCl) for *T. fragiferum* and from 26.8 (single species with inoculant from *T. fragiferum* without NaCl) to 70.5 (single species with inoculant from *T. repens* with NaCl) for *T. repens*. The average stolon length was the less variable morphological trait for *T. fragiferum*, with values from 21.5 to 37.5 cm; but for *T. repens* it varied from 14.0 (species coexistence with inoculant from *T. repens* with NaCl) to 40.5 cm (the treatment with the lowest number of stolons). 

In general, tissue water content had a tendency to be lower in *T. repens* in comparison to *T. fragiferum* (Table 4). The striking difference was in the case of species coexistence in the presence of inoculant from *T. repens*, where this combination resulted in low water content in *T. fragiferum* concomitant with increased water content in *T. repens*. However, NaCl treatment reversed this effect. There was a tendency for all NaCl treatments to induce higher tissue water content for *T. fragfierum*, but that was not the case for *T. repens*.

In respect to shoot fresh mass, ANOVA results indicated a significant effect of species coexistence, inoculant, and NaCl, but for shoot dry mass a significant effect was by inoculant and species (Table 5). Three significant two-way interactions were found: between species coexistence and NaCl, inoculant and species, and NaCl and species, for both fresh mass and dry mass. A significant three-way interaction between inoculant, NaCl, and species indicated different responses of *T. fragiferum* and *T. repens* shoot growth on inoculant type and NaCl treatment. Details on the relative effect of NaCl, inoculation, and species coexistence on shoot fresh and dry mass are shown in Table 6, Table 7 and Table 8. 

For *T. fragiferum*, NaCl treatment had a significant stimulative effect for both fresh and dry mass, except in the treatment with species coexistence and inoculant from *T. repens* where it had no effect (Table 6). The highest positive effect from NaCl treatment was for *T. fragiferum* plants with species coexistence, inoculated with bacteria isolated from the same species, reaching 205 and 188% for fresh mass and dry mass, respectively, as compared to respective controls without NaCl. For non-inoculated *T. repens* plants in single species conditions, NaCl treatment had a significant negative effect, but it was fully reversed by species coexistence. Inoculation of *T. repens* plants in single species conditions with bacteria isolated from either species also fully reversed the negative consequences of NaCl treatment on shoot growth. However, when *T. repens* plants inoculated with rhizobia from *T. fragiferum* were cultivated together with *T. fragiferum* plants, NaCl treatment led to a dramatic decrease in shoot growth. The results for growth of *T. repens* in species coexistence conditions, when plants were inoculated with rhizobia from the same species, were somehow contradictory, as no recovery from NaCl treatment was evident in respect to fresh mass but there was full recovery for a dry mass.

Inoculation of single species NaCl non-treated *T. fragiferum* plants with rhizobia from the same species resulted in significant growth stimulation (Table 7). The effect of rhizobia from *T. repens* was less pronounced and non-significant for fresh mass. When *T. fragiferum* plants without species coexistence were treated with NaCl, which itself stimulated shoot growth, the stimulative effect of inoculation was less pronounced for rhizobia from *T. fragiferum* with a non-significant effect for fresh mass, while in the case of rhizobia from *T. repens* it was stimulated for fresh mass and smoothed out for dry mass. For *T. fragiferum* plants in species coexistence conditions, the stimulative effect of rhizobial inoculation was less pronounced in the case of bacteria from *T. fragiferum* without NaCl treatment, but clearly more pronounced for all other treatment combinations. The highest positive effect was caused by inoculation with rhizobia from *T. repens* without NaCl treatment, where relative growth reached 188 and 280%, for fresh and dry mass, respectively, as compared to the respective controls without inoculant. Inoculation of *T. repens* plants with either inoculant without NaCl treatment resulted in a more pronounced stimulation of fresh mass accumulation than dry mass accumulation, while for NaCl-treated plants only dry mass accumulation was stimulated. Inoculation of *T. repens* plants in species coexistence conditions, in general, showed opposite effects on fresh and dry mass accumulation to those evident in single species conditions. There was no significant effect of inoculation on shoot dry mass accumulation except in treatment with rhizobia from *T. fragiferum* without NaCl. Shoot fresh mass increased in all treatment combinations, but the effect was not statistically significant for inoculation with rhizobia from *T. fragiferum* without NaCl.

Species coexistence had negative consequences for the growth of *T. fragiferum* without inoculation in treatment combinations both with and without NaCl as evident by the significant decrease in both fresh and dry mass (Table 8). The same level of growth inhibition of *T. fragiferum* by species coexistence was seen for the plants inoculated with rhizobia from *T. fragiferum* and without NaCl treatment, but NaCl treatment eliminated this negative effect. In the case of inoculation of *T. fragiferum* plants with rhizobia from *T. repens*, species coexistence had no effect on growth, or it was positive in the case of dry mass in the treatment without NaCl. For non-inoculated *T. repens* plants without NaCl treatment, species coexistence had no effect on growth, but the effect was positive in the case of NaCl treatment. *T. repens* plants inoculated with rhizobia from *T. fragiferum* positively responded to species coexistence when there was no NaCl addition, but in NaCl-treated plants there was no effect of species interaction for fresh mass, but the effect was negative for dry mass. In the case of *T. repens* plants inoculated with rhizobia from *T. repens*, species coexistence had no significant stimulative effect on shoot growth. Moreover, for dry mass in plants without NaCl treatment the effect was significantly negative.

### 3.4. Physiological Parameters

**Figure 3:**
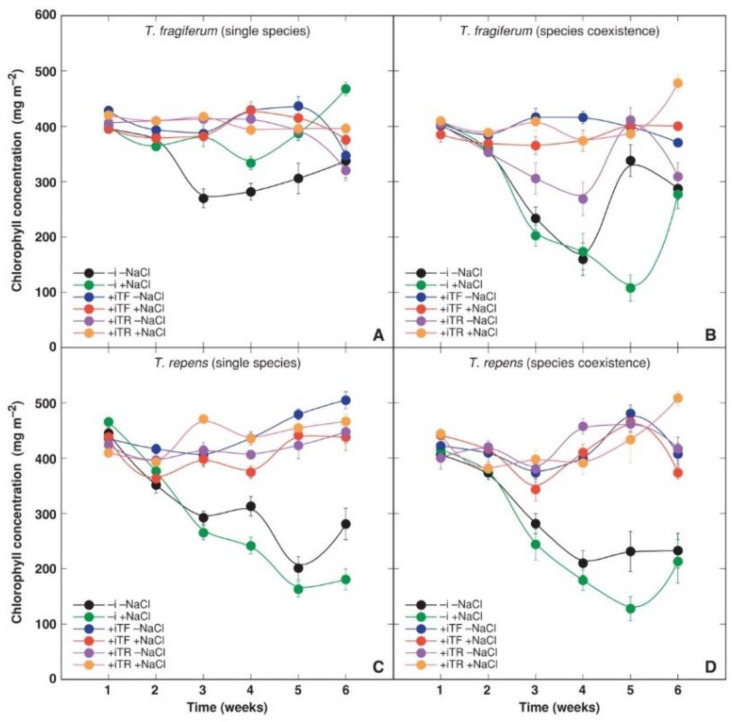
Time course of chlorophyll concentration in leaves of *T. fragiferum* in single species conditions (**A**), *T. fragiferum* in species coexistence conditions (**B**), *T. repens* in single species conditions (**C**), *T. repens* in species coexistence conditions (**D**). Each data point represents the mean from three randomly selected containers per treatment with three measurements per container ±SE. –i, without rhizobial inoculation; +iTF, inoculated with rhizobia from *T. fragiferum*; +TR, inoculated with rhizobia from *T. repens*.

Photosynthesis-related parameters leaf chlorophyll concentration and chlorophyll *a* fluorescence-related Performance Index were measured non-destructively during the experiment to follow physiological changes in plants due to various treatment combinations. Leaf chlorophyll concentration seemed to be a relatively stable parameter (Figure 3). ANOVA results indicated that leaf chlorophyll concentration was significantly affected by species coexistence and inoculant but not by NaCl (Table 9). Moreover, the parameter was species-specific and significantly changed with time. A number of significant two-way interactions were found: between species coexistence and inoculant, species coexistence and NaCl, inoculant and NaCl, inoculant and species, NaCl and time, species coexistence and time, and inoculant and time (Table 9). Highly significant three-way interactions were evident between species coexistence, inoculant, and NaCl; inoculant, NaCl, and species indicated that NaCl modified the effects of other factors and their interactions. 

The negative effect of the absence of inoculant on leaf chlorophyll concentration was less pronounced in *T. fragiferum* plants in single species conditions, with NaCl treatment partially abolishing this effect (Figure 3A). A more pronounced decrease in chlorophyll concentration in *T. fragiferum* plants due to the absence of bacterial inoculation was evident in conditions of species coexistence (Figure 3B). For these plants, also treatment with inoculant from *T. repens* resulted in a temporary decrease in chlorophyll concentration, but NaCl treatment completely abolished this effect. Recovery of chlorophyll concentration during the final part of the experiment indicated possible contamination of plants with rhizobia. In *T. repens*, the chlorophyll concentration was also negatively affected by the absence of rhizobia and this effect was relatively similar for plants grown in single species (Figure 3C) and species coexistence conditions (Figure 3D). 

**Figure 4:**
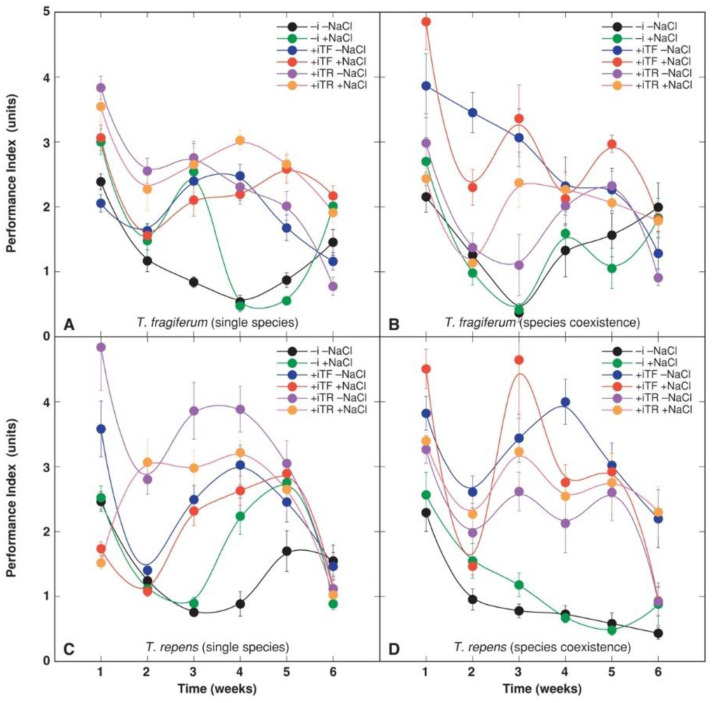
Time course of chlorophyll *a* fluorescence parameter Performance Index in leaves of *T. fragiferum* in single species conditions (**A**), *T. fragiferum* in species coexistence conditions (**B**), *T. repens* in single species conditions (**C**), *T. repens* in species coexistence conditions (**D**). Each data point represents the mean from three randomly selected containers per treatment with three measurements per container ±SE. –i, without rhizobial inoculation; +iTF, inoculated with rhizobia from *T. fragiferum*; +TR, inoculated with rhizobia from *T. repens*.

The time course of the Performance Index showed more variability in respect to species, timing, and different treatments (Figure 4). According to the results of ANOVA, Performance Index was significantly affected by inoculant, was species-specific, and changed with time (Table 9). As in the case of chlorophyll concentration, also for Performance Index, several two-way interactions were significant: between species coexistence and inoculant, species coexistence and NaCl, inoculant and NaCl, inoculant and species, NaCl and time, species coexistence and time, inoculant and time, and species and time. Among the three-way interactions, the one between species coexistence, inoculant, and time was more significant. 

For *T. fragiferum* plants in single species conditions, all rhizobial inoculation treatments showed an increased level of Performance Index, while the timing of the effect differed in dependence on the inoculant used (Figure 4A). NaCl treatment significantly increased the Performance Index in non-inoculated plants only for 3 weeks, but the effect was longer in the case of inoculation with rhizobia from *T. fragiferum* (4 weeks) and *T. repens* (5 weeks). Under species coexistence, inoculation with rhizobia from *T. repens* did not initially stimulate the Performance Index, but further, some positive effect was evident in NaCl-treated plants (Figure 4B). In contrast, plants responded positively to inoculation with rhizobia from *T. fragiferum.* In general, NaCl treatment had no significant effect on the Performance Index in species coexistence conditions.

Inoculation of *T. repens* plants with rhizobia had a significant initial stimulative effect on the Performance Index that was more pronounced in the case of inoculants from *T. repens* (Figure 4C). At this stage, NaCl treatment resulted in a significant decrease in the Performance Index of inoculated plants even below the level found in non-inoculated plants. With increasing substrate salinity, Performance Index values recovered up to the level found in respective treatments without NaCl addition. A positive effect of rhizobial inoculation was significant during the middle part of the experiment, after some pronounced decline of the parameter at week 2. Species coexistence had similar initial effects on the Performance Index of *T. repens* plants (Figure 4D) similar to those in *T. fragiferum* in species coexistence conditions. Plants inoculated with rhizobia from *T. fragiferum* and treated with NaCl had a higher level of Performance Index in the middle part of the experiment, but these inoculated with rhizobia from *T. repens* had a lower value, in comparison to conditions without species coexistence.

### 3.5. Ion Concentration

In *T. fragiferum* plants, treated with NaCl, the highest average concentration of Na^+^ accumulated in petioles, followed by stolons and leaves (Table 5). In NaCl-treated *T. repens* plants, the highest concentration of Na+ was found in petioles, followed by flower stalks, leaves, stolons and flowers. According to results of ANOVA, species had significant effect on Na^+^ concentration in all plant parts (Table 10). Concentration of Na^+^ was significantly lower in parts of *T. repens*, with a relative concentration of 38, 49 and 41%, in comparison to stolons, leaves and petioles, respectively, of *T. fragiferum*. Species coexistence significantly affected Na^+^ concentration only in stolons and petioles (Table 10). The effect in general was negative in all parts of *T. fragiferum*, but not always statistically significant. However, negative effect of species coexistence on accumulation of Na^+^ was less pronounced in *T. repens*. 

The highest average K^+^ concentration in *T. fragiferum* was found in petioles, followed by leaves and stolons (Table 11). For *T. repens* plants, K^+^ concentration decreased in the order: flowers > petioles > leaves > flower stalks > stolons. ANOVA results showed that no factor had a significant effect on K^+^ concentration in stolons and petioles, but in leaves, it was significantly affected by species coexistence and species (Table 12). *T. fragiferum* in single species conditions had a higher K^+^ concentration in leaves in comparison to that in *T. repens* in single species conditions. In several combinations, there was a significant effect of NaCl treatment on K^+^ concentration (Table 11). In leaves of *T. fragiferum*, K^+^ decreased in NaCl-treated plants with species coexistence (72 to 80% relative to respective NaCl non-treated plants) and in plants inoculated with rhizobia from *T. repens* in single species conditions (75%). In petioles of *T. fragiferum* negative effect of NaCl treatment on K^+^ concentration was found in plants in single species conditions (72 to 80%) and in plants without inoculant (73%) or inoculated with rhizobia from *T. repens* (74%) in species coexistence conditions. In contrast, a positive effect of NaCl treatment on K^+^ concentration prevailed in *T. repens* plants. The effect was especially pronounced in petioles and flower stalks, where NaCl treatment increased tissue K^+^ concentration in all combinations except plants without species coexistence, inoculated with rhizobia from *T. repens*. The effect ranged from 129 to 223% in flower stalks and 113 to 164% in petioles, in comparison to K^+^ concentration in non-treated plants. 

The leaf K^+^:Na^+^ concentration ratio, in general, was higher for *T. repens* than that for *T. fragiferum*, both in NaCl-treated and non-treated plants (Table 11).

### 3.6. Oxidative Processes and Tissue Damage

**Figure 5:**
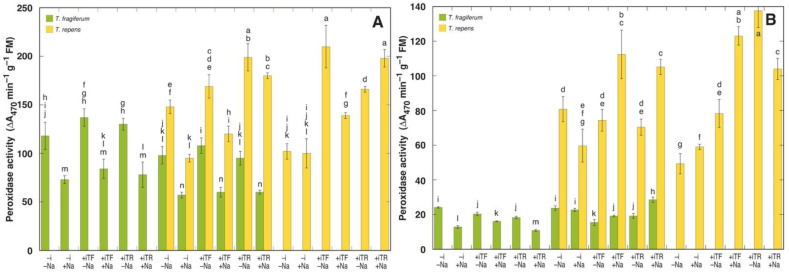
Peroxidase activity in leaves (**A**) and leaf petioles (**B**) of *T. fragiferum* and *T. repens* at the harvest. Data are the means from three replicates ± SE, the results are expressed on fresh mass basis. –i, without inoculant; +iTF, with inoculant isolated from *T. fragiferum*; +iTR, with inoculant isolated from *T. repens*; −Na, no NaCl added; +Na, NaCl up to 400 mM. Different letters indicate significant differences between the treatments (*p* < 0.05).

**Figure 6:**
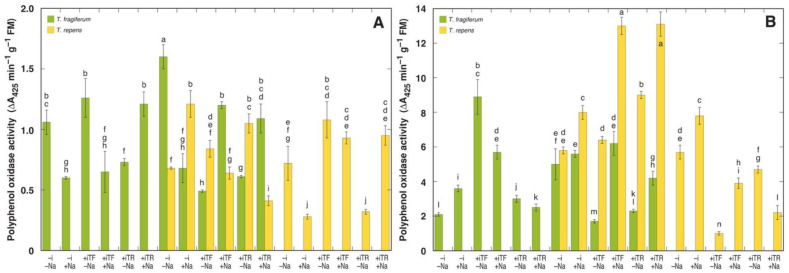
Polyphenol oxidase activity in leaves (**A**) and leaf petioles (**B**) of *T. fragiferum* and *T. repens* at the harvest. Data are the means from three replicates ± SE, the results are expressed on fresh mass basis. −i, without inoculant; +iTF, with inoculant isolated from *T. fragiferum*; +iTR, with inoculant isolated from *T. repens*; −Na, no NaCl added; +Na, NaCl up to 400 mM. Different letters indicate significant differences between the treatments (*p* < 0.05).

The relative intensity of enzymatic oxidative processes was assessed by means of measuring peroxidase (Figure 5) and polyphenol oxidase (Figure 6) activity in leaves and petioles of *T. fragiferum* and *T. repens* plants. In *T. fragiferum*, peroxidase activity in petioles was significantly lower than that in leaves, but this was not the case for *T. repens*. According to the results of ANOVA, peroxidase activity in leaves was significantly affected by all experimental factors, but the activity in petioles only by species coexistence and species (Table 13). When peroxidase activity in leaves was expressed on a dry mass basis, it was significantly affected only by inoculant and species (data not shown). The effect of NaCl treatment on peroxidase activity was significantly negative in leaves and petioles of *T. fragiferum* plants in single species conditions (Figure 5), with relative activity from 53 to 70 units, in comparison to respective non-treated plants. In conditions of species coexistence, leaf peroxidase activity of *T. fragiferum* also decreased by NaCl treatment (56 to 63%), but increased in petioles of plants inoculated with rhizobia (124 to 148%). In *T. repens*, NaCl treatment had a variable effect on peroxidase activity both in leaves and petioles. Without NaCl treatment, peroxidase activity in leaves of *T. fragiferum* was higher in single species conditions. Inoculation with rhizobia increased leaf peroxidase activity in *T. fragiferum* in single species conditions, but with species coexistence, this effect was seen only for rhizobia from *T. fragiferum*. Inoculation increased also leaf peroxidase activity in *T. repens*, but the effect was not statistically significant for rhizobia from *T. fragiferum*. 

Polyphenol oxidase activity in both species was variably affected by NaCl treatment in leaves as well as petioles (Figure 6). A significant effect was evident only in the case of the activity in petioles, which was affected by species.

**Figure 7:**
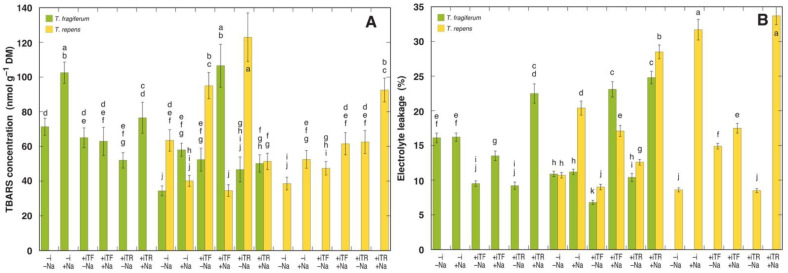
TBARS concentration in leaves (**A**) and electrolyte leakage (**B**) of *T. fragiferum* and *T. repens* at the harvest. Data are the means from three replicates ± SE, the results for TBARS are expressed on dry mass basis. −i, without inoculant; +iTF, with inoculant isolated from *T. fragiferum*; +iTR, with inoculant isolated from *T. repens*; −Na, no NaCl added; +Na, NaCl up to 400 mM. Different letters indicate significant differences between the treatments (*p* < 0.05).

Biochemical indicator of peroxidative membrane damage, TBARS concentration, showed relatively moderate variability between experimental treatments (Figure 7A). According to the ANOVA, no factor significantly affected TBARS concentration and no consistent effect of NaCl treatment was evident. However, TBARS concentration was significantly increased by NaCl in several treatment combinations: in *T. fragiferum* in single species conditions without rhizobial inoculation (by 44%) and with inoculant from *T. repens* (47%), in species coexistence without inoculation (69%) and with inoculant from *T. fragiferum* (103%); in *T. repens* in single species conditions without rhizobial inoculation (36%) and with inoculant from *T. fragiferum* (30%) and *T. repens* (48%). Species coexistence completely prevented an NaCl-induced increase in TBARS in leaves of *T. repens* (Figure 7A). TBARS concentration showed a relatively tight positive correlation with leaf Na^+^ concentration (*R^2^* = 0.6053) when two treatments were excluded, where high TBARS concentration was evident in *T. repens* plants inoculated with both types of rhizobia in species coexistence conditions but not treated with NaCl. When only NaCl-treated plants were considered, the tightness of the correlation increased (*R^2^* = 0.6815). 

Electrolyte leakage from plant tissues varied from 6.8 to 24.8% in *T. fragiferum* and from 8.5 to 33.7% in *T. repens* (Figure 7B). According to the results of ANOVA, the parameter was significantly affected only by NaCl treatment (Table 13). There was a significant increase in electrolyte leakage by NaCl in *T. fragiferum* plants inoculated by rhizobia, but no effect was seen in non-inoculated plants (Figure 7B). The highest increase in electrolyte leakage due to NaCl treatment was for *T. fragiferum* plants in species coexistence conditions, inoculated with rhizobia from *T. fragiferum* (340% in comparison to respective NaCl non-treated plants). NaCl treatment resulted in a significant increase in electrolyte leakage in all treatment combinations in *T. repens*, with the highest increase in plants in single species conditions, non-inoculated with rhizobia (369%) or inoculated with rhizobia from *T. repens* (397%). No significant correlation was found between TBARS concentration and tissue electrolyte leakage.

## 4. Discussion

### 4.1. Experimental System

In experiments where rhizobial symbiosis is excluded, special care must be considered as bacteria can easily disperse from inoculated containers and contaminate control containers [14]. In the present study, the use of sterilized substrate and materials—as well as individual watering system for containers—were precautions to minimize possible initial contamination. However, no particular actions were performed in the second part of the experiment to prevent touching between plant shoots. As a result, exclusion of rhizobial symbiosis was only temporal, as indicated by the presence of nodules on roots of non-inoculated plants at the end of the experiment (Ievinsh et al., unpublished results) as well as by characteristic increase in photosynthesis-related parameters 6–8 weeks after the start of the experiment (Figure 3 and Figure 4). Consequently, it is possible to suggest that differences in physiological responses of non-inoculated vs. inoculated plants at least during the first 5 weeks of the experiment can be considered as a result of the absence/presence of rhizobial symbiosis. 

Salinity level due to the application of increasing concentration of NaCl up to 400 mM in the present study was 230 to 290 mS m^−1^ (2.3 to 2.9 dS m^−1^), similar values were found in natural habitat (239 mS m^−1^). However, this is relatively low when compared to seasonal variation of electrical conductivity found in salinity-affected wetland soil where the two *Trifolium* species grow (1.3 to 25.5 dS m^−1^) [47]. In natural conditions, the salinity level has high temporal heterogeneity as a result of interaction between inundation with saline water and rain-dependent washing out of salts.

With respect to the possibilities and limitations of the model system used, it is important to emphasize that it was more realistic in respect to the multitude of interactions in natural conditions. Besides the already mentioned putative problems with rhizobial contamination during the experiment, the results obtained reflected a high degree of experimental complexity and were difficult to analyze. Therefore, substantial knowledge of physiology was necessary to discuss the outcome of the present experiment.

### 4.2. Species Coexistence and Response to Salinity

Interaction between plant species has been assessed mostly from the point of resource (light, water, nutrients) availability [33] sometimes analyzing also the effect of other environmental factors [48]. It is usually found that in relatively optimal conditions plants have a stronger tendency for competition due to faster resource acquisition, while in less optimal conditions cooperation will prevail [49,50]. However, in the case of interaction between individuals belonging to different species, self/non-self recognition can be an important additional factor [51]. Therefore, in the present study, we tried to find out how physiological characteristics are affected by the cocultivation of two individuals from different species in comparison to those of individuals from a single species, with a substrate salinity acting as a main environmental factor. 

The coexistence of species in natural conditions, including salt marshes, is manifested by both positive and negative interactions [52]. Within biotic interactions, competition is likely the most important factor for the establishment of zonation in salt marshes [53]. Moreover, it was demonstrated that a trade-off between the competitive ability of a species and its stress tolerance contributes to plant zonation in salt marshes and other plant communities [33], but physiological mechanisms of the competition are far from clear [54]. If two species have different tolerance to some environmental factor, a change of intensity of this factor could result in a shift of competition-related balance between the species. Therefore, it is logical to assume that the competitive ability of more salt-tolerant (or halophytic) species increases with an increase in soil salinity in further positively affecting its distribution [53]. In the present study, it was hypothesized that plants of potentially more salt-tolerant clover species *T. fragiferum* will have better physiological performance and growth in comparison to *T. repens* only if coexisting in conditions of elevated soil salinity. 

Based on the literature analysis, it was expected that *T. fragiferum* will show better salinity tolerance [5,55] as compared to *T. repens* [8]. Both plant species indeed differentially responded to NaCl treatment. For *T. fragiferum*, NaCl treatment significantly increased shoot biomass both in single species and species coexistence conditions except for dry mass in plants inoculated with rhizobia from *T. repens* in species coexistence (Figure 2). Increased shoot fresh mass in *T. fragiferum* by NaCl treatment was partially due to increased tissue water content (Table 4). It is well known that enhanced tissue water content as a result of salinity is a characteristic adaptive response of both halophytes and glycophytes [56] and could be related to increased salinity tolerance. In contrast, NaCl treatment resulted in a significant decrease in shoot biomass of *T. repens* in non-inoculated plants in single species conditions and, most severely, in plants inoculated with rhizobia from *T. fragiferum* during species coexistence (Figure 2). A contradictory effect of NaCl treatment was evident in respect to shoot fresh mass (decrease) and dry mass (no significant effect) for *T. repens* plants in species coexistence conditions inoculated with rhizobia from *T. repens*, showing that salinity induced a decrease in tissue water content in this treatment combination. Experiments with more realistic NaCl treatment, mimicking short-term seawater flooding conditions, have revealed that *T. repens* plants are negatively affected by soil flooding with saline water, but also indicated that the response may be ecotype-specific [57]. 

Species coexistence had a significant negative relative effect on the growth of *T. fragiferum* for non-inoculated plants and plants inoculated with rhizobia from *T. fragiferum* but only without NaCl (Table 4). Significant coexistence-dependent growth stimulation of *T. fragiferum* was evident only for dry mass in plants inoculated with rhizobia from *T. repens* and cultivated without NaCl. In contrast, species coexistence benefited *T. repens* plants under NaCl treatment without bacterial inoculation and plants without NaCl treatment but inoculated with rhizobia from *T. fragiferum* (Table 4). A significant negative effect of species coexistence on the growth of *T. repens* was evident for plants inoculated with rhizobia from *T. repens* without NaCl and for plants inoculated with rhizobia from *T. fragiferum* with NaCl. Both morphological and physiological data confirmed that *T. repens* is a more dominant species than *T. fragiferum*, but the dominance was significantly decreased by NaCl treatment and inoculation with rhizobia that in part could be related to nitrogen-dependent changes in competition characteristics between legume species [14]. Besides competition for resources, the negative effect of species coexistence could be related also to the production and release of allelopathic compounds [58].

The most striking effect of species coexistence as affected by rhizobial inoculant was seen in the presence of rhizobia from *T. repens*, where shoot water content decreased in *T. fragiferum* and increased for *T. repens* (Table 4). The effect was clearly due to species coexistence, as it was not evident in single species conditions. The addition of NaCl fully reversed this hydration-related effect. No similar relationship was seen in the case of inoculant from *T. fragiferum*, pointing to some specific characteristics of rhizobial isolates, disappearing in conditions of salinity.

Returning to the initial hypothesis, namely, that rare species *T. fragiferum* can outcompete more common species *T. repens* only in conditions of increased substrate salinity, it appeared that NaCl treatment indeed had a higher positive effect on the growth of *T. fragiferum* in conditions of species coexistence, but the positive effect of bacterial inoculant in coexisting species, especially by rhizobia isolated from *T. repens*, was significantly more pronounced. We did not directly compare responses of single individual plants with those of two individuals of either species (species coexistence). However, preliminary experiments showed that single individuals of either *T. fragiferum* or *T. repens* responded to substrate salinity differentially in comparison to two individuals of the same species (Ievinsh et al., unpublished results). Thus, the physiological response of the individual changed in respect to the identity of the coexisting individual; e.g., the outcome of the response of the individual to environmental cues is predicted by the interaction between species.

Possible physiological mechanisms of more pronounced salinity tolerance in *T. fragiferum*, based on the present results, need to be discussed in more detail. The reduction in root to shoot transport of Na^+^ is described as one of the mechanisms related to increased NaCl tolerance, at least, for glycophytic species [59]. In the present study, the compartmentation of Na^+^ in different parts of shoots of *T. fragiferum* and *T. repens* was evident, with a higher accumulation of the ion in leaf petioles. For *T. repens*, Na^+^ was excluded from flowers, accumulating in flower stalks. NaCl-treated *T. fragiferum* plants had significantly higher concentrations of Na^+^ in all studied plant parts in comparison to *T. repens* (Table 10). Consequently, *T. repens* exhibited a typical feature of partial Na^+^ exclusion from shoots, while *T. fragiferum* accumulated Na^+^ in shoots, representing a higher degree of cellular tolerance possibly due to efficient Na^+^ compartmentation in cell vacuoles [60] and by means of the active antioxidant system [61]. 

K^+^:Na^+^ homeostasis is considered to be one of the major traits for salinity tolerance, at least, for glycophyte species [62]. In the present study, NaCl treatment had a significant effect on K^+^ concentration in leaves in some treatment combinations, but in general, variation in the K^+^:Na^+^ concentration ratio between treatments was not associated with observed differences in plant growth. It seems that at least *T. fragiferum* can efficiently replace K^+^ with Na^+^ for maintenance of cellular osmotic functions, as already noted in previous studies with potential halophytic species [32].

The decrease in leaf chlorophyll concentration and photosynthesis, including photochemical efficiency of photosystem II, is a well-documented response of plants to suboptimal NaCl treatment [63,64]. In a study with *T. repens*, it was found that no decrease in maximum quantum yield of photosystem II (Fv/Fm) by NaCl treatment up to 100 mM was evident [65]. No general direct effect of NaCl on photosynthesis-related characteristics was found in the present study but NaCl treatment significantly affected the effect of other factors on these parameters (Table 9). 

In a study with a halophytic plant species *Cakile maritima*, it was shown that salinity-induced a decrease in plant photosynthetic activity that further led to decreased growth and reproduction [66]. Quantum yield of photosystem II of *C. maritima* increased at optimum salinity (100 mM NaCl) but was significantly decreased at 300 and 500 mM NaCl. In the present study, the Performance Index was not much affected by the gradual NaCl increase in the substrate. For rhizobia-inoculated *T. fragiferum* plants under single species conditions, the Performance Index significantly increased by NaCl treatment only at the end of the experiment, but this effect was not evident under species coexistence (Figure 2). For *T. repens* under single species conditions, non-inoculated plants exhibited a temporal increase in the Performance Index by NaCl treatment, but for plants inoculated with rhizobia from *T. repens*, the Performance Index level temporarily decreased in NaCl-treated plants.

While biological factors are a less studied type of environmental influence, it is logical to presume that for legume species symbiosis with nitrogen-fixing rhizobial bacteria is a critical factor in interspecific interactions. In a microcosm study with a number of dune grassland species, it was established that three of the four studied legume species require rhizobial symbiosis for successful coexistence with non-legume species [14]. This aspect will be discussed further (Section 4.3).

It should be noted also that in natural conditions of a salt-affected meadow, *T. fragiferum* plants have a significant level of arbuscular mycorrhizal colonization in roots, reaching both high frequency (99%) and intensity (68%) [47]. In experiments with *T. repens*, it has been shown that the presence of arbuscular mycorrhizal symbiosis can modify how clonal integration affects plant performance in heterogeneous environmental conditions [67]. Arbuscular mycorrhizal symbiosis is shown to be important also for salinity tolerance [68]. Consequently, in natural conditions, not only rhizobial symbiosis but also mycorrhiza can have a significant impact on the result of the coexistence of allied species.

### 4.3. Bacterial Inoculation

Inoculation with native symbiosis-forming N_2_-fixing bacteria was a significant factor in the present study affecting the outcome of the interaction between *T. fragiferum* and *T. repens*. In nutrient-limiting conditions, rhizobial inoculation usually has a positive effect on the growth of clover species and other legume plants, depending on both the compatibility and efficiency of used bacterial strains [69]. Indeed, both fresh and dry mass of *Trifolium* plant shoots increased in all treatment combinations inoculated with rhizobia (Figure 2). However, it is difficult to unequivocally interpret the mineral availability status of plants in the present experiments. First, both nitrate-N and ammonia-N in equal amounts were present in the fertilizer used here. Both total amount and balance between different chemical forms of N can significantly affect plant growth and development in a species-specific manner [70,71], as well as the rhizobial symbiosis of legume species including clover [72]. At least, the use of equal proportions of both forms of N allowed to exclude any specific effects associated with the use of a particular chemical form of N. Second, nitrogen availability and its chemical forms itself regulates salt tolerance in plants [73]. 

Direct comparison of nitrogen-fixation efficiency of different *Trifolium* species by the two specific isolates of rhizobia was outside the scope of the present study. However, based solely on growth data, it is possible to assume that general specificity to a particular rhizobial isolate was comparably low. In single species conditions, the accumulation of shoot dry matter of both *T. fragiferum* and *T. repens* was not statistically different between treatments with either rhizobia (Figure 2B). The identity of bacterial isolate could affect the fate of species interaction, as, in contrast to what was expected, inoculation with rhizobia from *T. repens* in *T. fragiferum* plants in species coexistence conditions without NaCl treatment resulted in a two-fold increase in shoot dry matter in comparison to inoculation with rhizobia from *T. fragiferum* (Figure 2B). In the respective treatments, *T. repens* plants exhibited a two-fold decrease in shoot dry matter. In conditions of coexistence of different legume species, an individual of one species can benefit from nitrogen fixed by an individual from another species due to more efficient rhizobial symbiosis of the latter, similar to what has been described for interaction between legume and non-legume species [74]; or due to more salt-tolerant symbiotic bacteria. It is important to note that the presence of NaCl completely abolished the mentioned effect. It is usually thought that nodule-forming rhizobia are more salinity tolerant than their host plants [75], but it is clear from the present results that differences in salinity tolerance of rhizobial strains can have a significant effect on the outcome of species coexistence in conditions of a salt marsh. Yet, functional aspects of rhizobial symbiosis are highly sensitive to salinity, with nitrogen fixation being more vulnerable in comparison to ammonium assimilation [76].

It appears that the nitrogen-fixing ability of rhizobia isolated from *T. repens* was significantly more pronounced in comparison to that from *T. fragiferum*, allowing *T. fragiferum* plants to benefit from rhizobial symbiosis in *T. repens* plants in conditions of species coexistence, but this needs to be examined further. Specificity of rhizobia could be a less important aspect in rhizobia–clover interaction in natural conditions, as compared to differences in the efficiency of particular strains and by different bacteria–clover combinations [69]. A comparative study from a subtropical region showed a large diversity in respect to nodule-forming species in roots of *T. fragiferum* un *T. repens*: both plants can form nodules with bacterial species from genera *Bradyrhizobium* and *Rhizobium* (predominantly strains of *Rhizobium leguminosarum*), but *T. repens* plants can also have symbiosis with *Sinrhizobium* species, while *T. fragiferum* with *Mesorhizobium* species [77]. Moreover, it is not clear whether the particular inoculant consisted only of one rhizobial strain.

Several mechanisms have been proposed for the beneficial effect of bacterial inoculants in conditions of increased soil salinity. Tissue-specific regulation of the Na^+^ transporter by a soil bacterium *Bacillus subtilis* has been shown to represent a critical mechanism for the maintenance of low Na^+^ in *Arabidopsis thaliana* in further conferring increased NaCl tolerance [78]. This mechanism is likely to be effective also in the case of *B. subtilis*-induced salt tolerance of wheat [79]. Inoculation of *T. repens* with *Azospirillum brasilense*, a free-living soil bacterium, increased the leaf chlorophyll concentration of plants both in control (by 62%) and low salinity (64, 75, and 61%, in 40, 80, and 120 mM NaCl, respectively) conditions [80]. In addition, inoculation of *T. repens* with *A. brasilense* significantly reduced Na^+^ accumulation in shoots and roots in low salinity conditions [80]. In the present study, tissue Na^+^ concentration decreased by rhizobial inoculation in leaves and stolons of NaCl-treated *T. fragiferum* plants in single species conditions but increased by inoculation in leaves and stolons of *T. repens* plants except in leaves of single species plants inoculated with rhizobia from *T. fragiferum* (Table 10). A relatively close correlation between Na^+^ concentration and a potential indicator of peroxidative membrane damage, TBARS concentration, was evident, indicating that a decrease in tissue Na^+^ concentration could be associated with less tissue damage. 

Some additional evidence for rhizobial symbiosis-dependent protection against NaCl could be derived from analysis of peroxidase activity in leaves where bacterial inoculation with either rhizobia resulted in a significant increase in relative peroxidase activity. This increase was relatively higher for *T. repens* in single species conditions. 

### 4.4. Evaluation of Cellular Damage and Physiological Performance

Among biochemical indicators of cellular damage, measurement of malondialdehyde (MDA) concentration in tissues by means of the analysis of TBARS concentration has been widely used as an indicator of the relative degree of lipid peroxidation due to unfavorable conditions leading to endogenous oxidative stress [81]. In particular, salinity usually increases tissue MDA concentration in sensitive species and cultivars but not in resistant ones [82]. For a halophytic species *Atriplex portulacoides*, no significant increase in leaf MDA concentration was seen even at 1000 mM NaCl salinity [63]. During the comparison of two accessions of halophyte *Cakile maritima*, it was found that growth stimulation of the more salt-tolerant accession occurred at 100 mM NaCl, together with decreased MDA concentration, 66% lower than that of control plants [61]. In the present study, there were statistically significant differences in TBARS concentration in leaves of both *Trifolium* species between different treatments (Figure 7). While, according to the ANOVA results, there was no general trend of effect on TBARS concentration by any of the experimental factors, this parameter was significantly increased by NaCl treatment in several treatment combinations. Moreover, when only NaCl-treated plants were considered, the leaf TBARS concentration relatively tightly positively correlated with leaf Na^+^ concentration. Most interestingly, the coexistence of *T. repens* with *T. fragiferum* completely eliminated the stimulative effect of NaCl treatment on TBARS concentration in *T. repens*, which was evident in single species conditions (Figure 7). In *T. repens* plants not treated with NaCl, high TBARS concentration in leaves was evidently caused by other factors, i.e., coexistence with *T. fragiferum* together with rhizobial inoculation. 

Relative electrolyte leakage in leaves of a legume *Glycine soja* plants (10%) increased up to 30% by 150 mM NaCl treatment [44], which is in a range of changes documented also in the present study. Electrolyte leakage was highly significantly affected by NaCl treatment in the present study (Table 13). It is usually thought that electrolyte leakage in NaCl-treated plants is a direct consequence of the effect of Na on membrane permeability [64], but no significant correlation between TBARS concentration and electrolyte leakage was evident. During the comparison of MDA concentration and degree of membrane leakage in Zea mays plants subjected to salinity, it was concluded that permeability characteristics of membranes could be affected by factors other than lipid peroxidation [83]. Furthermore, it was argued that electrolyte leakage from senescing leaves does not represent a reliable characteristic of cell membrane damage [84]. As no inhibition of photochemical efficiency of photosystem II was caused by NaCl treatment in spite of the significant increase in TBARS concentration in several treatment combinations, it seems that in the conditions of the present experiment NaCl treatment did not result in a significant increase in oxidative damage to chloroplastic macromolecular structures over some basal level. Consequently, recorded changes in growth between different treatment combinations were not associated with deleterious effects at the level of tissue damage but rather were reflections of physiological changes due to species coexistence, NaCl treatment, rhizobial inoculation, and interactions between them.

It is usually found that salinity-induced increase in peroxidase activity is a characteristic response of more tolerant species indicating activation of defense mechanisms [85]. Thus, salt-tolerant species *Plantago maritima* showed a significant increase in leaf peroxidase activity at 100 mM NaCl (no decrease in growth), while salt-sensitive species *Plantago media* (53% decrease in dry matter) had no response at this salinity [86]. However, when time-course analysis was performed, it became evident that peroxidase activity increases in salt-tolerant species occurred only at early stages after the start of the treatment (reflecting induction of defense responses), but for sensitive species, more late increase reflects an enzymatic response to cellular damage [87]. In the present study, peroxidase activity was significantly affected by NaCl treatment, decreasing in leaves of *T. fragiferum*, but the particular effect of treatment on peroxidase activity in *T. repens* plants depended on the combination of factors (presence or absence of particular rhizobial inoculant as well as single species conditions vs. species coexistence; Figure 5). It is possible that the effect of NaCl on peroxidase activity was at least partially due to stimulation of increased tissue water content by the treatment, which was more pronounced in *T. fragiferum* (Table 1). According to ANOVA analysis, when leaf peroxidase activity was expressed on a dry mass basis, it was significantly affected only by inoculant and species, in comparison to the significant effect of all factors in the case when the activity was expressed on a fresh mass basis (Table 13).

However, while NaCl resulted in decreased activity of polyphenol oxidase in several treatment combinations, there was no uniform response of the enzyme activity to NaCl treatment that might be attributed to indirect (or interactive) effects of salinity on this enzyme. Studies on the effect of abiotic factors besides tissue wounding on polyphenol oxidase activity are relatively scarce. It was shown that an increase in polyphenol oxidase activity in leaves of *Trifolium pratense* correlated with the degree of cell damage due to cattle grazing [88]. 

Decreased growth in the absence of rhizobial inoculation occurred together with a decrease in leaf chlorophyll concentration (Figure 3) as well as a lowered photochemical capacity of photosystem II as indicated by chlorophyll *a* fluorescence measurements (Figure 4). Total leaf chlorophyll concentration is often correlated to N nutrition, as a decrease in N tissue availability can result in inhibited chlorophyll synthesis [89]. It seems that the characteristic decrease in leaf chlorophyll concentration in both *T. fragiferum* and *T. repens* plants in the absence of rhizobial inoculation reflects some common adaptive mechanism of clover species to nitrogen-fixing rhizobia, where active symbiosis is necessary to maintain high rates of chlorophyll synthesis. Similar to the present results, inoculation with rhizobia of *Phaseolus lunatus* plants led to a higher leaf chlorophyll content in comparison to uninoculated, nitrogen-supplied plants [90]. Inoculation with both types of rhizobia resulted in the early increase in the Performance Index in both studied species (Figure 4). The highest initial increase for both *Trifolium* species was caused by rhizobia from *T. repens* in single species conditions and that from *T. fragiferum* in species coexistence conditions. It is not known though to what extent depression of photochemical activity in non-inoculated plants in the present study can be a consequence of possible N shortage.

## 5. Conclusions

During the coexistence of allied species, the interaction between biotic and abiotic factors can affect the outcome of the species coexistence, as significant two-way interactions (between species coexistence and NaCl, inoculant and species, and NaCl and species) and a significant three-way interaction (between inoculant, NaCl and species) on plant shoot growth of *Trifolium* species were found in the present study. NaCl treatment was an important positive factor for *T. fragiferum*, resulting in better growth in conditions of species coexistence, but the positive effect of bacterial inoculant, especially, by rhizobia isolated from *T. repens*, was significantly more pronounced. A decrease in peroxidase activity in leaves was a good indicator of relative NaCl tolerance, while the absence/presence of rhizobial inoculation was reflected by changes in leaf chlorophyll concentration, and, more importantly, by characteristics of the photochemical activity of photosystem II. For NaCl-treated plants, leaf TBARS concentration relatively tightly positively correlated with leaf Na^+^ concentration. Consequently, one of the reasons why *T. fragiferum* is exclusively associated with the sea coast in its Northern range of distribution is related to a higher competitive ability with allied species in conditions of increased substrate salinity, based on better physiological salinity tolerance. Though it remains to be an open question as to why the competitive ability or adaptive capacity of *T. fragiferum* is higher in its main range of distribution where no specific relationship with saline habitats is found.

## Figures and Tables

**Table 1:** Experimental treatments used in the present study.

Treatment	*T. fragiferum*	*T. repens*	iTF	iTR	NaCl
TFss–i–NaCl	+++	–	–	–	–
TFss–i+NaCl	++	–	–	–	+
TFss+iTF–NaCl	++	–	+	–	–
TFss+iTF+NaCl	++	–	+	–	+
TFss+iTR–NaCl	++	–	–	+	–
TFss+iTR+NaCl	++	–	–	+	+
TFTR–i–NaCl	+	+	–	–	–
TFTR–i+NaCl	+	+	–	–	+
TFTR+iTF–NaCl	+	+	+	–	–
TFTR+iTF+NaCl	+	+	+	–	+
TFTR+iTR–NaCl	+	+	–	+	–
TFTR+iTR+NaCl	+	+	–	+	+
TRss–i–NaCl	–	++	–	–	–
TRss–i+NaCl	–	++	–	–	+
TRss+iTF–NaCl	–	++	+	–	–
TRss+iTF+NaCl	–	++	+	–	+
TRss+iTR–NaCl	–	++	–	+	–
TRss+iTR+NaCl	–	++	–	+	+

ss, single species; –i, without bacterial inoculant; +iTF, with inoculant from *T. fragiferum*; +iTR, with inoculant from *T. repens*; –NaCl, no NaCl added; +NaCl, NaCl up to 400 mM.

**Table 2:** Changes of substrate electrical conductivity (EC, mS m^−1^) during the experiment.

Treatment	Week 2	Week 4	Week 6
TFss–i–NaCl	89.5 ± 5.7 ef	71.4 ± 2.7 e	53.3 ± 3.6 gh
TFss–i+NaCl	119.9 ± 10.3 abc	163.9 ± 14.6 bcd	252.4 ± 8.8 cd
TFss+iTF–NaCl	107.0 ± 5.4 cd	68.3 ± 4.9 ef	53.5 ± 8.8 gh
TFss+iTF+NaCl	132.6 ± 4.4 a	148.1 ± 8.0 d	235.7 ± 9.1 de
TFss+iTR–NaCl	87.4 ± 6.8 ef	70.1 ± 2.4 e	50.1 ± 1.8 h
TFss+iTR+NaCl	112.3 ± 5.5 bcd	145.9 ± 8.9 d	230.8 ± 9.2 e
TFTR–i–NaCl	81.7 ± 6.6 f	58.1 ± 2.3 g	50.1 ± 2.1 h
TFTR–i+NaCl	115.3 ± 3.0 bc	183.6 ± 6.3 ab	270.7 ± 8.1 abc
TFTR+iTF–NaCl	129.8 ± 11.8 a	76.6 ± 3.6 e	71.4 ± 5.4 f
TFTR+iTF+NaCl	121.5 ± 7.4 ab	181.1 ± 5.3 ab	255.7 ± 24.4 abcd
TFTR+iTR–NaCl	94.9 ± 3.7 ef	65.7 ± 3.3 efg	58.2 ± 1.1 g
TFTR+iTR+NaCl	105.7 ± 3.8 cd	156.5 ± 7.4 cd	262.7 ± 5.0 bc
TRss–i–NaCl	93.8 ± 5.0 ef	60.6 ± 2.3 fg	55.2 ± 4.2 gh
TRss–i+NaCl	103.9 ± 4.9 de	190.7 ± 11.0 a	289.5 ± 6.6 a
TRss+iTF–NaCl	113.5 ± 6.1 bcd	56.6 ± 2.9 g	55.1 ± 1.5 gh
TRss+iTF+NaCl	123.5 ± 6.4 ab	173.5 ± 6.6 abc	264.7 ± 12.7 abc
TRss+iTR–NaCl	105.2 ± 8.2 cd	61.3 ± 2.0 fg	52.7 ± 2.5 h
TRss+iTR+NaCl	133.5 ± 4.9 a	177.5 ± 8.0 ab	273.3 ± 7.4 ab

Data are the means from five replicates with four measurements per container per treatment in each time point. Different letters within a column indicate significant differences between the treatments (*p* < 0.05). ss, single species; –i, without bacterial inoculant; +iTF, with inoculant from *T. fragiferum*; +iTR, with inoculant from *T. repens*; −NaCl, no NaCl added; +NaCl, NaCl up to 400 mM.

**Table 3:** ANOVA analysis of substrate electrical conductivity.

Source of Variation	df	Mean Square	*F*
Species coexistence	2	3394	12.81 ***
Inoculant	2	1046	3.95 *
NaCl	1	797 795	3012.24 ***
Time	2	59684	225.35 ***
Species coexistence × inoculant	4	804	3.04 *
Species coexistence × NaCl	2	3506	13.24 ***
Inoculant × NaCl	2	2165	8.18 ***
Species coexistence × time	4	489	1.85
Inoculant × time	4	1661	6.27 ***
NaCl × time	2	193 783	731.67 ***
Species coexistence × inoculant × NaCl	4	414	1.56
Residuals	240	265	

* *p* < 0.05, *** *p* < 0.001.

**Table 4:** Morphological characteristics of *T. fragiferum* and *T. repens* plants grown in initially sterile soil.

Treatment	H_2_O Content (g g^−1^ DM)	Number of Leaves	Number of Stolons	Total Stolon Length (mm)	Average Stolon Length (mm)
*T. fragiferum*					
TFss–i–NaCl	4.9	736 ± 92 bcd	44.3 ± 5.7 def	1097 ± 136 ef	25.0 ± 2.3 defg
TFss–i+NaCl	5.8	829 ± 89 abc	55.3 ± 6.4 cd	1465 ± 122 b	27.5 ± 2.3 cdef
TFss+iTF–NaCl	4.2	607 ± 75 def	52.0 ± 6.0 cde	1516 ± 174 b	30.0 ± 3.5 bcd
TFss+iTF+NaCl	4.5	835 ± 99 ab	57.5 ± 5.0 bc	2084 ± 189 a	36.5 ± 3.8 ab
TFss+iTR–NaCl	4.3	725 ± 13 cd	42.3 ± 4.8 efg	1087 ± 32 ef	23.3 ± 1.8 fg
TFss+iTR+NaCl	5.4	697 ± 77 cd	37.0 ± 5.6 f	1352 ± 151 bcd	37.5 ± 2.5 a
TFsc–i–NaCl	4.3	345 ± 37 i	33.5 ± 5.5 g	714 ± 31 g	21.5 ± 2.5 gh
TFsc–i+NaCl	4.7	585 ± 5 ef	34.5 ± 3.5 f	1111 ± 83 def	33.0 ± 3.0 abc
TFsc+iTF–NaCl	4.7	539 ± 20 fg	54.5 ± 2.5 cd	1456 ± 48 b	26.5 ± 1.5 defg
TFsc+iTF+NaCl	5.2	478 ± 11 gh	42.0 ± 5.0 efg	1363 ± 156 bcd	32.5 ± 0.5 bc
TFsc+iTR–NaCl	2.5	510 ± 75 fg	32.5 ± 4.5 g	1097 ± 95 ef	34.0 ± 2.7 ab
TFsc+iTR+NaCl	5.3	939 ± 130 ab	77.0 ± 11.0 a	2255 ± 182 a	29.5 ± 0.5 cd
*T. repens*					
TRss–i–NaCl	3.5	599 ± 48 def	27.0 ± 3.4 h	656 ± 75 g	24.0 ± 2.2 efg
TRss–i+NaCl	3.5	385 ± 16 hj	31.8 ± 2.8 h	775 ± 71 g	25.0 ± 3.5 defg
TRss+iTF–NaCl	3.3	652 ± 16 de	26.8 ± 2.8 h	1049 ± 98 ef	40.5 ± 5.5 a
TRss+iTF+NaCl	3.2	687 ± 69 cd	40.3 ± 4.3 fg	1106 ± 96 def	27.5 ± 0.5 def
TRss+iTR–NaCl	3.3	556 ± 49 efg	45.0 ± 3.9 def	1176 ± 102 cde	26.0 ± 2.8 defg
TRss+iTR+NaCl	3.4	861 ± 77 ab	70.5 ± 6.9 ab	1560 ± 112 b	22.0 ± 2.9 fgh
TRsc–i–NaCl	3.0	435 ± 78 ghi	38.5 ± 4.5 fgh	641 ± 78 g	16.0 ± 3.0 hi
TRsc–i+NaCl	3.3	486 ± 68 fgh	27.5 ± 2.5 h	776 ± 12 g	29.0 ± 3.0 cde
TRsc+iTF–NaCl	4.2	962 ± 108 a	57.5 ± 6.5 bc	1626 ± 178 b	27.5 ± 3.8 cdef
TRsc+iTF+NaCl	3.8	817 ± 94 abc	63.5 ± 6.6 abc	1478 ± 172 b	22.0 ± 3.5 fgh
TRsc+iTR–NaCl	6.4	773 ± 79 abc	40.5 ± 3.5 fg	957 ± 12 f	24.0 ± 2.0 efg
TRsc+iTR+NaCl	3.7	394 ± 31 hi	39.0 ± 4.6 fgh	516 ± 98 g	14.0 ± 2.5 i

Data are the means from five replicates ± SE, the results are expressed on a single plant basis. TF, *T. fragiferum*; TR, *T. repens*; ss, single species; sc, species coexistence; –i, without inoculant; +iTF, with inoculant isolated from *T. fragiferum*; +iTR, with inoculant isolated from *T. repens*; –NaCl, no NaCl added; +NaCl, NaCl up to 400 mM. Different letters within a column indicate significant differences between the treatments (*p* < 0.05).

**Table 5:** ANOVA analysis of shoot mass parameters.

Source of Variation	df	Shoot Fresh Mass			Shoot Dry Mass		
		Mean Square	*F*	ω^2^	Mean Square	*F*	ω^2^
Species coexistence Inoculant NaCl	2	3053	13.58 ***	0.078	8.22	0.73	–0.002
	2	6233	27.73 ***	0.165	243.16	21.68 ***	0.188
	1	3680	16.37 ***	0.048	25.10	2.24	0.006
Species Species coexistence × inoculant	1	6	0.03	–0.003	72.27	6.44 *	0.025
	4	375	1.37	0.008	10.00	0.89	–0.002
Species coexistence × NaCl Inoculant × NaCl Inoculant × species NaCl × species	2	2385	10.61 ***	0.059	43.39	3.87 *	0.026
	2	184	0.82	–0.001	0.04	0.00	–0.009
	2	916	4.08 *	0.019	225.46	20.10 ***	0.174
	1	16133	71.79 ***	0.219	71.09	6.34 *	0.024
Species coexistence × inoculant × NaCl	4	173	0.77	–0.03	5.35	0.48	–0.009
Inoculant × NaCl × species Residuals	2	1718	7.64 ***	0.041	52.35	4.67 *	0.033
	96	225			11.22		

* *p* < 0.05, *** *p* < 0.001.

**Table 6:** Relative effect of NaCl (values from the respective treatment combinations without NaCl were indicated as 100%) on total fresh and dry mass of shoots of the two *Trifolium* species.

Treatment	Relative Mass (%)							
	*T. fragiferum*				*T. repens*			
	Single Species		Species Coexistence		Single Species		Species Coexistence	
	FM	DM	FM	DM	FM	DM	FM	
–i	171 ± 6 *	147 ± 13 *	183 ± 28 *	168 ± 24 *	75 ± 6 *	75 ± 4 *	102 ± 13	93 ± 6
+iTF	132 ± 6 *	125 ± 9 *	205 ± 22 *	188 ± 23 *	98 ± 6	100 ± 5	54 ± 3 *	59 ± 4 *
+iTR	151 ± 4 *	125 ± 6 *	176 ± 19 *	96 ± 14	104 ± 4	101 ± 5	71 ± 17 *	112 ± 27

The data are derived from Table 4. Significant differences (*p* < 0.05) from the respective control without NaCl are indicated by *.

**Table 7:** Relative effect of inoculation with two types of rhizobia (values from the respective treatment combinations without inoculant were indicated as 100%) on total fresh and dry mass of shoots of the two *Trifolium* species.

Treatment	Relative Mass (%)							
	*T. fragiferum*				*T. repens*			
	Single Species		Species Coexistence		Single Species		Species Coexistence	
	FM	DM	FM	DM	FM	DM	FM	
–NaCl, iTF	145 ± 5 *	161 ± 7 *	148 ± 15 *	138 ± 10 *	200 ± 8 *	126 ± 7 *	119 ± 4	207 ± 14 *
+NaCl, iTF	114 ± 5	138 ± 10 *	166 ± 17 *	155 ± 19 *	106 ± 5	168 ± 8 *	156 ± 9 *	96 ± 7
–NaCl, iTR	117 ± 7	130 ± 9 *	188 ± 31 *	280 ± 19 *	151 ± 21 *	127 ± 8 *	121 ± 4 *	81 ± 14

The data are derived from Table 4. Significant differences (*p* < 0.05) from the respective control without inoculant are indicated by *.

**Table 8:** Relative effect of species coexistence (values from the respective treatment combinations in single species conditions were indicated as 100%) on total fresh and dry mass of shoots of the two *Trifolium* species.

Treatment	Relative Mass (%)							
	*T. fragiferum*				*T. repens*			
	–NaCl		+NaCl		–NaCl		+NaCl	
	FM	DM	FM	DM	FM	DM	FM	
–i	61 ± 6 *	67 ± 6 *	65 ± 9 *	77 ± 11 *	98 ± 15	111 ± 13	134 ± 17 *	138 ± 9 *
+iTF	62 ± 6 *	58 ± 4 *	97 ± 10	87 ± 11	164 ± 7 *	134 ± 13 *	91 ± 4	79 ± 6 *
+iTR	98 ± 16	145 ± 10 *	112 ± 13	112 ± 16	122 ± 17	71 ± 13 *	83 ± 19	78 ± 19

The data are derived from Table 4. Significant differences (*p* < 0.05) from the respective control without species coexistence are indicated by *.

**Table 9:** ANOVA analysis of leaf chlorophyll concentration and Performance Index.

Source of Variation	df	Chlorophyll Concentration		Performance Index	
		Mean Square	*F*	Mean Square	*F*
Species coexistence	2	186357	64.96 ***	1.02	1.40
Inoculant	2	1594961	555.991 ***	179.57	247.36 ***
NaCl	1	1211	0.42	1.58	2.18
Species	1	89559	31.22 ***	3.02	4.16 *
Time	5	139103	48.49 ***	64.53	88.89 ***
Species coexistence × inoculant	4	116126	40.48 ***	29.43	40.53 ***
Species coexistence × NaCl	2	22439	7.82 ***	18.63	25.66 ***
Inoculant × NaCl	2	52265	18.22 ***	2.86	3.94 *
Inoculant × species	2	34570	12.05 ***	12.85	17.70 ***
NaCl × species	1	8278	2.89	0.02	0.03
NaCl × time	5	23032	8.03 ***	2.65	3.66 **
Species coexistence × time	10	17178	5.99 ***	2.61	3.59 ***
Inoculant × time	10	153539	53.52 ***	12.37	17.04 ***
Species × time	5	5382	1.88	4.24	5.84 ***
Species coexistence × inoculant × NaCl	4	31668	11.04 ***	3.61	4.98 ***
Inoculant × NaCl × species	2	23868	8.32 ***	2.73	3.76 *
Residuals	1237	2869		0.73	

* *p* < 0.05, ** *p* < 0.01, *** *p* < 0.001.

**Table 10:** Tissue Na^+^ concentration (g kg^−1^ DM) in different parts of *T. fragiferum* and *T. repens* at the harvest.

Treatment	Stolons	Leaves	Petioles	Flowers	Flower Stalks
*T. fragiferum*					
TFss–i–NaCl	0.93 ± 0.16 d	0.49 ± 0.06 f	0.71 ± 0.11 de	−	–
TFss–i+NaCl	12.75 ± 0.25 a	10.08 ± 0.08 a	19.83 ± 1.33 b	−	–
TFss+iTF–NaCl	0.78 ± 0.05 e	0.56 ± 0.01 f	0.61 ± 0.04 e	–	–
TFss+iTF+NaCl	9.75 ± 1.42 b	9.92 ± 0.92 ab	19.17 ± 0.83 b	–	–
TFss+iTR–NaCl	0.99 ± 0.33 d	0.66 ± 0.06 e	0.88 ± 0.15 d	–	–
TFss+iTR+NaCl	10.25 ± 1.08 b	8.58 ± 0.42 bc	23.25 ± 0.42 a	–	–
TFsc–i–NaCl	0.80 ± 0.35 e	0.73 ± 0.23 e	0.73 ± 0.21 de	–	–
TFsc–i+NaCl	7.38 ± 0.71 c	6.41 ± 1.36 d	17.22 ± 0.94 c	–	–
TFsc+iTF–NaCl	0.96 ± 0.24 d	0.82 ± 0.19 e	0.99 ± 0.17 d	–	–
TFsc+iTF+NaCl	7.13 ± 1.70 c	7.92 ± 0.25 cd	17.17 ± 0.94 c	–	–
TFsc+iTR–NaCl	1.04 ± 0.01 d	0.67 ± 0.07 e	0.83 ± 0.07 d	–	–
TFsc+iTR+NaCl	9.58 ± 0.42 b	7.08 ± 0.25 d	19.08 ± 0.08 b	−	–
*T. repens*					
TRss–i–NaCl	0.19 ± 0.02 e	0.41 ± 0.08 f	0.42 ± 0.10 d	0.40 ± 0.07 d	0.27 ± 0.08 g
TRss–i+NaCl	3.63 ± 1.37 ab	6.08 ± 0.58 a	11.33 ± 1.83 a	0.70 ± 0.12 c	2.12 ± 0.74 d
TRss+iTF–NaCl	0.25 ± 0.08 e	0.49 ± 0.08 ef	0.51 ± 0.01 d	0.37 ± 0.06 d	0.45 ± 0.05 f
TRss+iTF+NaCl	4.32 ± 0.92 a	3.09 ± 1.71 bc	5.87 ± 1.24 b	1.57 ± 0.88 b	5.24 ± 0.74 b
TRss+iTR–NaCl	0.32 ± 0.07 e	0.45 ± 0.08 f	0.44 ± 0.08 d	0.32 ± 0.12 de	0.37 ± 0.18 fg
TRss+iTR+NaCl	4.97 ± 0.53 a	7.02 ± 1.65 a	14.92 ± 1.92 a	1.83 ± 0.10 a	7.83 ± 0.76 a
TRsc–i–NaCl	0.65 ± 0.17 d	1.09 ± 0.44 d	0.25 ± 0.05 e	0.20 ± 0.04 e	0.27 ± 0.05 g
TRsc–i+NaCl	1.47 ± 0.27 c	0.69 ± 0.12 e	2.08 ± 0.75 c	0.49 ± 0.17 d	1.31 ± 0.28 e
TRsc+iTF–NaCl	0.27 ± 0.12 e	0.41 ± 0.13 f	0.41 ± 0.13 d	0.34 ± 0.06 d	0.52 ± 0.08 f
TRsc+iTF+NaCl	2.72 ± 0.38 b	2.56 ± 0.64 c	6.03 ± 0.93 b	0.80 ± 0.12 bc	3.60 ± 0.44 c
TRsc+iTR–NaCl	0.14 ± 0.03 f	0.28 ± 0.02 g	0.27 ± 0.07 e	0.27 ± 0.05 e	0.23 ± 0.04 g
TRsc+iTR+NaCl	4.17 ± 0.30 ab	5.19 ± 1.14 ab	7.42 ± 0.92 b	1.89 ± 0.21 a	6.13 ± 0.53 b

Data are the means from three replicates ± SE. TF, *T. fragiferum*; TR, *T. repens*; ss, single species; sc, species coexistence; −i, without inoculant; +iTF, with inoculant isolated from *T. fragiferum*; +iTR, with inoculant isolated from *T. repens*; −NaCl, no NaCl added; +NaCl, NaCl up to 400 mM. Different letters within a column indicate significant differences between the treatments (*p* < 0.05).

**Table 11:** Tissue K^+^ concentration (g kg^−1^ DM) in different parts of *T. fragiferum* and *T. repens* at the harvest and leaf K^+^/Na^+^ concentration ratio.

Treatment	Stolons	Leaves	Petioles	Flowers	Flower Stalks	Leaf K^+^:Na^+^ Ratio
*T. fragiferum*						
TFss–i–NaCl	6.24 ± 1.93 a	5.33 ± 0.17 c	10.50 ± 1.00 ab	−	–	10.88
TFss–i+NaCl	4.38 ± 0.41 ab	6.00 ± 0.30 ab	8.42 ± 0.25 d	–	–	0.60
TFss+iTF–NaCl	2.33 ± 0.10 d	5.83 ± 0.33 abc	9.92 ± 0.92 bc	–	–	10.41
TFss+iTF+NaCl	2.71 ± 0.51 cd	5.23 ± 0.27 c	8.33 ± 0.35 d	–	–	0.53
TFss+iTR–NaCl	3.95 ± 1.22 ab	6.67 ± 0.33 a	11.08 ± 0.42 ab	–	–	10.11
TFss+iTR+NaCl	3.46 ± 0.16 bc	4.99 ± 0.18 cd	7.92 ± 0.42 d	–	–	0.58
TFsc–i–NaCl	4.11 ± 1.72 ab	5.42 ± 0.75 bc	8.22 ± 1.28 cd	–	–	7.43
TFsc–i+NaCl	2.84 ± 0.34 cd	4.20 ± 0.65 d	6.02 ± 0.48 e	–	–	0.66
TFsc+iTF–NaCl	3.11 ± 0.15 bcd	6.89 ± 0.95 ab	8.61 ± 0.39 cd	–	–	7.18
TFsc+iTF+NaCl	3.22 ± 0.92 bcd	4.96 ± 0.04 cd	8.58 ± 0.08 d	–	–	0.63
TFsc+iTR–NaCl	3.83 ± 1.06 ab	6.25 ± 0.25 ab	11.67 ± 0.50 a	–	–	9.33
TFsc+iTR+NaCl	3.34 ± 0.18 bc	5.00 ± 0.83 cd	8.58 ± 0.92 cd	−	–	0.71
*T. repens*						
TRss–i–NaCl	4.07 ± 0.35 bc	6.92 ± 0.79 ab	7.75 ± 0.08 c	9.50 ± 0.56 ab	4.15 ± 0.10 e	16.88
TRss–i+NaCl	3.73 ± 0.58 bc	7.67 ± 0.67 a	10.08 ± 0.92 a	9.00 ± 0.68 bc	7.00 ± 0.14 c	1.26
TRss+iTF–NaCl	2.58 ± 0.48 def	6.17 ± 0.17 c	8.33 ± 0.33 b	10.42 ± 0.42 a	4.88 ± 0.34 d	12.59
TRss+iTF+NaCl	1.74 ± 0.40 f	6.89 ± 0.49 abc	9.39 ± 0.78 ab	8.25 ± 0.08 bcd	6.83 ± 0.67 c	2.23
TRss+iTR–NaCl	2.48 ± 0.27 def	6.75 ± 0.58 abc	6.92 ± 0.25 d	8.67 ± 0.67 bcd	5.15 ± 0.68 d	15.00
TRss+iTR+NaCl	2.37 ± 0.27 def	7.58 ± 0.08 a	6.67 ± 0.67 d	7.67 ± 0.43 d	4.88 ± 0.67 d	1.08
TRsc–i–NaCl	4.87 ± 0.81 ab	7.26 ± 0.71 ab	6.71 ± 0.63 d	8.17 ± 0.56 cd	8.00 ± 7.25 b	6.67
TRsc–i+NaCl	2.72 ± 0.30 de	6.51 ± 0.58 bc	9.00 ± 0.67ab	9.35 ± 0.60 bc	10.29 ± 1.48 ab	9.44
TRsc+iTF–NaCl	2.20 ± 0.40 ef	6.69 ± 0.50 abc	6.29 ± 0.44 d	8.08 ± 0.96 cd	3.18 ± 0.35 f	16.32
TRsc+iTF+NaCl	3.30 ± 0.51 cd	6.50 ± 0.38 bc	10.33 ± 0.89 a	7.67 ± 0.44 d	4.98 ± 0.22 d	2.54
TRsc+iTR–NaCl	2.08 ± 0.36 ef	6.17 ± 0.50 c	5.05 ± 0.28 e	8.00 ± 0.65 cd	4.49 ± 0.06 de	22.04
TRsc+iTR+NaCl	4.92 ± 0.22 a	7.36 ± 0.14 a	9.75 ± 0.58 a	7.83 ± 0.42 d	10.00 ± 0.77 a	1.42

Data are the means from three replicates ± SE. TF, *T. fragiferum*; TR, *T. repens*; ss, single species; sc, species coexistence; –i, without inoculant; +iTF, with inoculant isolated from *T. fragiferum*; +iTR, with inoculant isolated from *T. repens*; −NaCl, no NaCl added; +NaCl, NaCl up to 400 mM. Different letters within a column indicate significant differences between the treatments (*p* < 0.05).

**Table 12:** ANOVA analysis of tissue Na^+^ and K^+^ concentration.

Source of Variation	df	Na^+^ Concentration						K^+^ Concentration	
		Stolons		Leaves		Petioles		Leaves	
		Mean Square	*F*	Mean Square	*F*	Mean Square	*F*	Mean Square	*F*
Species Coexistence	2	1.91	13.07 ***	0.40	1.33	0.81	8.17 **	0.072	5.04 *
Inoculant	2	0.02	0.17	0.04	0.13	0.17	1.68	0.003	0.20
NaCl	1	34.93	239.22 ***	30.00 ***	100.78 ***	55.90	564.71 ***	0.021	1.50
Species	1	3.94	26.99 ***	1.95 *	6.55 *	4.31	43.52 ***	0.15	10.56 **
Residuals	17	0.15		0.30		0.10		0.014	

The data were log-transformed before analysis. * *p* < 0.05, ** *p* < 0.01, *** *p* < 0.001.

**Table 13:** ANOVA analysis of oxidative enzyme activity and tissue electrolyte leakage.

		Peroxidase Activity				Polyphenol Oxidase Activity		Electrolyte Leakage	
		Leaves		Petioles		Petioles		Leaves	
		Mean Square	*F*	Mean Square	*F*	Mean Square	*F*	Mean Square	*F*
Species Coexistence	2	4090	6.53 **	4.14	42.71 ***	17.88	2.71	38.0	1.44
Inoculant	2	3343	5.34 *	0.09	0.89	1.12	0.17	48.1	1.82
NaCl	1	7995	12.77 **	0.00	0.00	17.02	2.61	737.0	27.87 ***
Species	1	15586	24.89 ***	5.54	57.20 ***	76.60	11.74 **	10.3	0.39
Residuals	17	626		0.10		6.53		26.4	

* *p* < 0.05, ** *p* < 0.01, *** *p* < 0.001.

## Data Availability

All data reported here is available from the authors upon request.
